# Number of remaining teeth and its association with socioeconomic status in South Korean adults: Data from the Korean National Health and Nutrition Examination Survey 2012-2013

**DOI:** 10.1371/journal.pone.0196594

**Published:** 2018-05-10

**Authors:** Yang Hyun Kim, Kyungdo Han, David Vu, Kyung-Hwan Cho, Sang Hwa Lee

**Affiliations:** 1 Department of Family Medicine, Korea University College of Medicine, Seoul, Republic of Korea; 2 Department of Medical Statistics, Catholic University College of Medicine, Seoul, Republic of Korea; 3 Wing Dental Center, Alberta, Canada; 4 Department of Dentistry, St. Paul’s Hospital College of Medicine, The Catholic University of Korea, Seoul, Republic of Korea; Universidad Miguel Hernandez de Elche, SPAIN

## Abstract

**Background:**

Socioeconomic status (SES) is associated with systemic disease and influences oral and general health. Several studies have found inequalities associated with oral health and SES. We examined the relationship between tooth loss and SES in Korean adults using data from the 2012–2013 Korean National Health and Nutrition Examination Survey. Methods: A total of 7,005 participants were included in this study. Subjects were divided into two groups depending on their total number of natural teeth: <20 and ≥20. Next, participants were divided into quartiles depending on household income and educational level. Multivariate logistic regression was used to obtain odds ratios (OR) for remaining teeth according to income and education levels.

**Results:**

As income and education levels increased, subjects were more likely to have ≥20 remaining teeth (p-value and p-value for trend <0.001), brush their teeth more than three times per day, use extra oral products, and have regular oral-health checkups (all p<0.001). The odds of having ≥20 remaining teeth increased with increases in income and education, after adjusting for all covariates (OR = 1.493 for income Q3, OR = 1.571 for income Q4; OR = 1.763 for 10–12 years education, OR = 2.189 for ≥13years education).

**Conclusion:**

Subjects with higher SES had more remaining teeth than subjects with lower SES. Preserving remaining teeth should be encouraged in subjects with low SES by promoting good oral-health behavior and encouraging more oral-health checkups.

## Introduction

Tooth loss is a common condition resulting from dental caries, periodontal disease, trauma, or extraction during orthodontic treatment [1]. Previous studies found that tooth loss was associated with systemic conditions, such as metabolic syndrome [2, 3], dementia [4], obesity [5], renal disease [6], cardiovascular disease [7, 8], diabetes mellitus [9], and certain types of cancer [10], in addition to other dental problems such as periodontitis.

In general, socioeconomic status (SES) is associated with the aforementioned systemic diseases [11–14]. Subjects with low SES have higher prevalence of obesity, coronary heart disease, and cancer than high SES subjects [11–14]. These people are also more likely to have poor oral and general health behaviors [15–17]. SES is associated with inequalities in oral health and subjects with low SES had higher rates of tooth loss than high SES subjects [18–20]. However, there are some controversies regarding the relationship between SES and tooth loss [21–23] because most of previous studies used data from countries with ethnically heterogeneous population or only adjusted for a few variables, such as age, gender, and area of residence. Specifically, there have been no many studies with nationwide representative data in an ethnically homogeneous population such as Korea.

Korean National Health and Nutrition Examination Survey (KNHANES) is a nationwide cross-sectional survey conducted by the South Korean Ministry of Health and Welfare using a representative sample of the entire Korean population obtained by cluster sampling design. Korea is a country characterized with a relatively homogeneous Asian population in terms of ethnicity that permits exclusion of racial/ethnical biases in the examination of the relationship between SES and number of remaining teeth excluding. In this study we examined the relationship between tooth loss and SES in Korean adults using a representative sample from the 2012–2013 KNHANES.

## Materials and methods

### Survey overview

Data from the 2012–2013 KNHANES was used in this study. The KNHANES includes national level health and nutrition data collected from surveys and physical examinations [24]. KHANES participants were stratified into multiple stages, prorated by age from the 2005 National Census Registry, and selected using a cluster sampling design to obtain a representative sample of non-institutionalized civilians of both sexes from whole geographic regions of South Korea Face-to-face interviews were carried out by trained interviewers.

### Subjects

This paper used the data from KNHANES a nationwide cross-sectional survey conducted by the South Korean Ministry of Health and Welfare. Survey procedure was used to account for the complex sampling design and to provide approximations of the entire Korean population. Moreover, for the representative sampling area of each region, the stratification and cluster are sampled and extracted to represent the whole Korean population. The participants visited the examination center and trained agents and dentists surveyed the study’s representative population of South Koreans using well-made questionnaires, and performed physical examinations.

A total of 21,811 subjects (12,417 men and 13,487 women) aged 19 years or older were included in the KNHANES. First, we only included subjects who were older than 40 years (n = 8,714). We excluded subjects who had insufficient data (n = 1,709). Ultimately, 7,005 subjects were included in this study. The study protocol was approved by the Institutional Review Board of the Korean Center for Disease Control and Prevention (2012-01EXP-01-2C, and 2013-07CON-03-4C) and adheres to the ethical principles for medical research involving human subjects as defined by the Declaration of Helsinki. Written informed consent was obtained from all participants.

### Sociodemographic variables

Subjects completed a self-administered questionnaire about age, sex, family income, and education level. Household income was corrected for the number of family members and divided into quartiles. Education level was also divided into quartiles equal or less than 6years (≤6), 7 to 9 years (7–9), 10 to 12 years (10–12), and equal or more than 13 years (≥13) of education. These quartiles represent elementary school, middle school, high school and university respectively. Occupational status was also surveyed.

### General health behaviors

General health behaviors were also surveyed. We defined current smokers as those who currently smoked and those who had cumulatively smoked more than 100 cigarettes in their life as National Health Interview Survey (NHIS) concept [25]. Heavy alcohol intake was defined as more than three glasses a day. Physical activity was defined using the International Physical Activity Questionnaire [26], and regular exercise was defined as taking exercising more than three times per week at an intense level for more than 20 minutes per session or as exercising for more than 30 minutes per session.

### Anthropometric measurements

Height (cm) and weight (kg) were recorded by trained examiners to the nearest 0.1 cm and 0.1 kg, respectively, with the subjects in light clothing and without shoes. After a normal expiration, waist circumference (WC) was measured to the nearest 0.1 cm on a horizontal plane at the midpoint level between the iliac crest and the costal margin. Body mass index was obtained by dividing the subjects’ weight (kg) by the square of their height (m^2^).

### Definition of metabolic syndrome

According to the American Heart Association/National Heart, Lung, and Blood Institute’s Scientific Statement for Asians [27] the conditions for metabolic syndrome are met when three or more of the following criteria are present: WC ≥90 cm in men and ≥80 cm in women, fasting blood glucose level ≥100 mg/dL or use of antidiabetic medication, blood pressure ≥130/85 mmHg or use of antihypertensive medication, fasting triglyceride level ≥150 mg/dL or use of antidyslipidemic medication, high density lipoprotein cholesterol level <40 mg/dL in men and <50 mg/dL in women or use of antidyslipidemic medication.

### Oral-health behaviors

Subjects recorded the time(s) of the day when they brushed their teeth and whether they used extra oral products other than a manual tooth brush and tooth paste. In this survey extra oral products include dental floss, interdental brushes, mouthwash, and electric toothbrushes. The time of day was divided into the following periods: before or after breakfast, lunch, dinner, after snacks, and before bedtime. The frequency of toothbrushing was calculated as the number of toothbrushing events per day. Completion of an oral examination during the last 12 months was surveyed using self-questionnaires.

#### Number of remaining teeth and periodontitis

The subjects were surveyed about whether they had lost any of their natural teeth. We also counted the number of remaining teeth. Periodontitis status was diagnosed when the community periodontal index (CPI) was greater than or equal to “code 3,” which means that at least one dental site had a greater than 3.5-mm periodontal pocket, as per the definition set by the World Health Organization. The numbers of index teeth were 11, 16, 17, 26, 27, 31, 36, 37, 46, and 47. The appropriate CPI probe was used by trained dentists in accordance with the World Health Organization guidelines [28]. The inter-examiner mean Kappa value was 0.89 (0.55–1.00) [29].

### Statistical analyses

To analyze their general characteristics, subjects were divided into two groups depending on their total number of natural teeth: The group 1: the 20 or more teeth group (≥20), and the group 2: the less than 20 teeth group (<20). According to many other studies, having at least 20 natural teeth is required for the satisfactory function and esthetics [30–32].

The data were presented as either means ± standard errors (SE) for continuous variables or as percentages (SE) for categorical variables. We classified four education levels: 6 or less years of education (elementary school (≤6)), 7 to 9 years of education (middle school (7–9)), 10 to 12 years of education (high school (10–12)), and 13 or more years of education (≥13), The economic levels were divided in four (Q1, Q2, Q3, and Q4) We analyzed the remaining number of teeth groups with education and economic quartiles using Chi-square tests and general linear modeling. Oral-health behaviors according to SES quartile were analyzed with Chi-square tests. Using analysis of variance tests, the relationships between mean number of remaining teeth and income and education levels were analyzed in three different adjusted models. Model 1 was adjusted for age and sex. Model 2 was adjusted for age, sex, BMI, current smoking status, alcohol consumption habits, regular exercise, frequency of toothbrushing per day, use of extra oral hygiene products, presence of periodontitis, occupational status, and presence of metabolic syndrome. Model 3 was adjusted for the covariates in model 2 plus education or income levels. Multivariate logistic regression was applied to obtain odds ratios (OR) and 95% confidence intervals (CI) for the ≥20 teeth group according to income and education levels after adjusting for age, sex, BMI, smoking, alcohol intake, physical activity, periodontitis, occupation, frequency of tooth brushing, metabolic syndrome, income and educational levels. Because of our survey procedure and complex sampling design we used SAS version 9.2 (SAS Institute Inc., Cary, NC, USA) to estimate risks and associations for the entire Korean population. All the statistical tests were two-tailed, and a p<0.05 was considered statistically significant.

## Results

General characteristics of subjects are shown in Table 1. The group with fewer than 20 remaining teeth (group 2) present significantly higher average age, mean WC, lower rates of alcohol consumption, physical activity and employment (all p<0.05) than the group with 20 or more remaining teeth (group 1). Additionally, the percentage of subjects with metabolic syndrome, periodontitis, lower income (Q1), and lower educational level (≤6 years) were higher in the group 2 than in the group 1 (all p<0.05). The number of toothbrusing time per day, remaining teeth and percentage of subject with receiving regular oral examination and regular exercise were significantly higher in the group 1 than group 2. (all p<0.05).

**Table 1:** Subjects’ general characteristics according to number of remaining teeth.

	Number of remaining teeth (n)		
	<20	≥20	*p*
Unweighted (n)	4,056	2,949	
Age (yr)	67.6±0.4	53.3±0.2	<0.001
Men % (SE)	45.6 (1.6)	48.1 (0.6)	0.172
Body mass index (kg/m^2^)	24.2±0.1	24.1±0.1	0.558
Waist circumference (cm)	84.0±0.4	82.0±0.2	<0.001
Current smoking % (SE)	17.7 (1.4)	18.9 (0.7)	0.491
Heavy alcohol intake % (SE)	5.7 (0.9)	8.4 (0.5)	0.020
Regular exercise % (SE)	12.4 (1.2)	17.6 (0.6)	<0.001
Income % (SE)	`		<0.001
Q1	43.4 (2.0)	15.2 (0.8)	
Q2	27.5 (1.6)	25.4 (0.9)	
Q3	18.2 (1.6)	27.1 (0.9)	
Q4	10.9 (1.1)	32.2 (1.2)	
Education % (SE)			<0.001
≤6 yr	59.9 (1.8)	21.6 (0.9)	
7–9 yr	16.2 (1.2)	14.2 (0.6)	
10–12 yr	17.3 (1.3)	37.9 (0.9)	
13≤ yr	6.7 (0.9)	26.3 (1.1)	
Occupation % (SE)	42.7 (1.8)	66.6 (0.9)	<0.001
Number of remaining teeth (n)	12.3±0.2	26.1±0.4	<0.001
Frequency of toothbrushing/day % (SE)			<0.001
≤1	21.4 (1.4)	11.4 (0.6)	
2	43.7 (1.6)	40.4 (0.8)	
3	34.9 (1.7)	48.2 (0.9)	
Oral health checkup % (SE)	16.8 (1.3)	29.1 (0.8)	<0.001
Metabolic syndrome % (SE)	52.5 (1.7)	34.5 (0.8)	<0.001
Periodontitis % (SE)	42.8 (2.0)	35.0 (1.1)	<0.001

**Fig 1:**
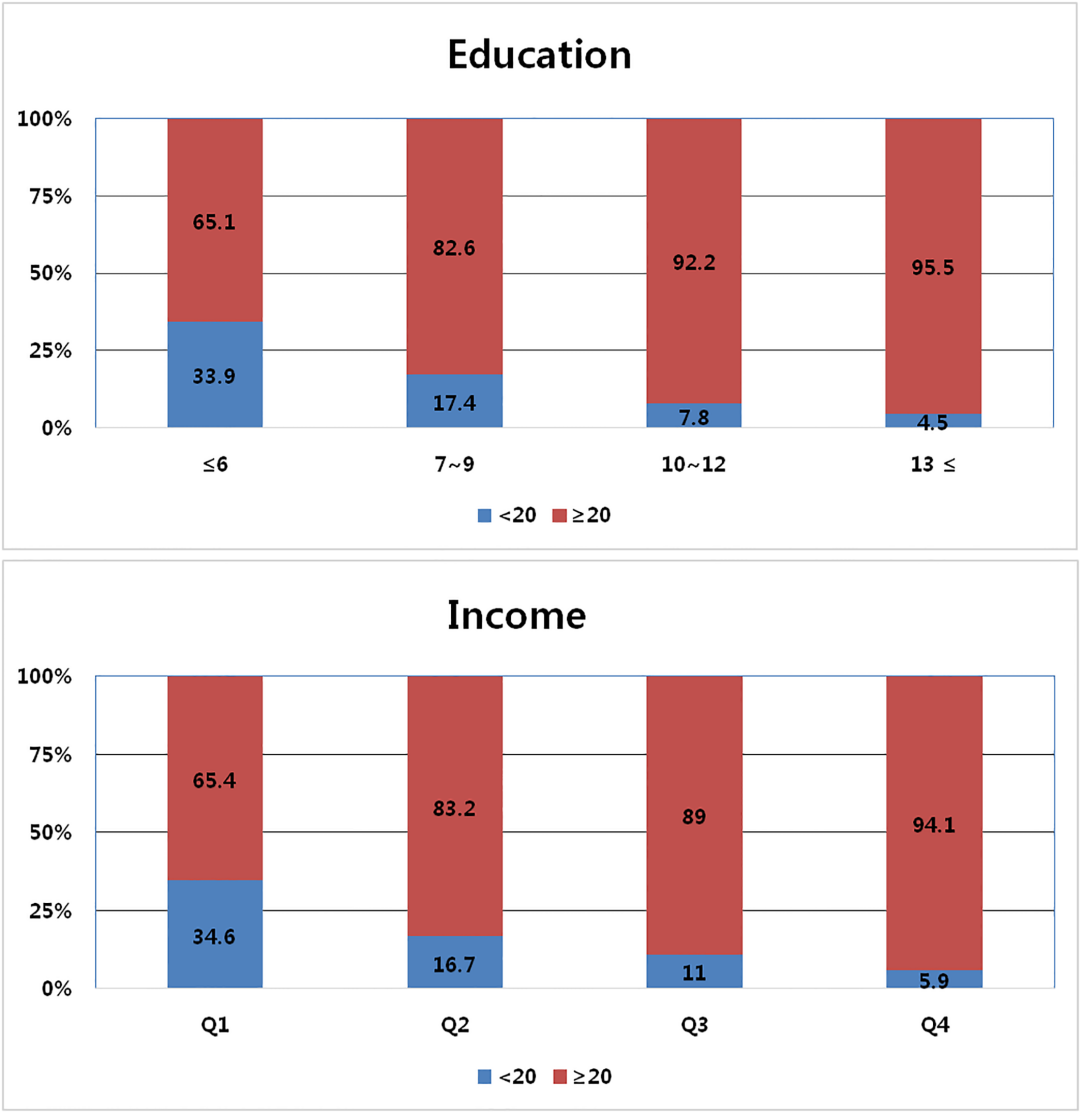
Remaining-teeth group proportions of income and education quartiles. Both p<0.001. Both p for trend <0.001.

Fig 1 shows the proportion of the two teeth groups according to the four income and education levels. As income and education levels increased, the proportion of subjects in the ≥20 teeth group increased while the <20 teeth group decreased (all p-values and p for trends <0.001).

As income and educational levels increased, the proportion of subjects who brushed their teeth 2 or less times per day decreased, while the proportion of subjects who brushed their teeth more than 2 times per day increased (both p<0.001). The number of subjects who used extra oral hygiene products and received regular oral-health examinations also increased as income and educational levels increased (all p<0.001) (Tables 2 and 3).

**Table 2:** Oral health behaviors as proportions of income quartiles.

	Income (quartile)				
	Q1	Q2	Q3	Q4	*p*
Frequency of toothbrushing/day % (SE)					<0.001
≤ 1	19.4(1.4)	14.0(1.1)	11.6(1.0)	8.9(0.9)	
2	45.2(1.5)	41.2(1.4)	42.7(1.4)	36.2(1.4)	
≥ 3	35.5(1.5)	44.8(1.5)	45.7(1.5)	54.9(1.5)	
Use of extra oral products % (SE)	29.1(1.5)	43.1(1.5)	50.0(1.6)	57.2(1.5)	<0.001
Oral health checkup % (SE)	16.4(1.2)	23.9(1.3)	27.8(1.3)	36.6(1.3)	<0.001

**Table 3:** Oral health behaviors as proportions of educational levels.

	Education (year)				
	≤6	7–9	10–12	≥13	*p*
Frequency of toothbrushing/day % (SE)					<0.001
≤ 1	21.0(1.2)	15.6(1.5)	10.1(0.8)	6.2(0.8)	
2	47.8(1.2)	45.1(1.8)	38.5(1.2)	33.9(1.5)	
≥ 3	31.2(1.1)	39.3(1.9)	51.4(1.3)	59.9(1.6)	
Use of extra oral products % (SE)	28.0(1.2)	37.5(1.7)	54.3(1.3)	61.3(1.6)	<0.001
Oral health checkup % (SE)	14.0(0.9)	27.6(1.8)	28.6(1.1)	40.2(1.5)	<0.001

Table 4 shows the mean number of remaining teeth according to income and education levels. After adjusting for all covariates, as income and education increased, the number of remaining teeth increased (p<0.001 and 0.003, respectively, and p for trend <0.001).

**Table 4:** Mean number of remaining teeth according to income and education quartiles.

	Model 1	Model 2	Model 3
Income (quartile)			
Q1	22.2±0.2	22.6±0.2	22.8±0.2
Q2	22.9±0.2	23.3±0.2	23.3±0.2
Q3	23.5±0.1	23.8±0.1	23.8±0.1
Q4	24.0±0.1	24.2±0.1	24.1±0.1
*p*	<0.001	<0.001	<0.001
Education (year)			
≤6	22.3±0.2	22.7±0.2	22.9±0.2
7~9	23.7±0.1	24.0±0.1	23.8±0.1
10~12	23.2±0.2	23.5±0.2	23.5±0.2
13≤	24.0±0.1	24.0±0.1	23.8±0.2
*p*	<0.001	<0.001	0.003

Model 1 was adjusted for age and sex.

Model 2 was adjusted for age, sex, BMI, current smoking, alcohol drinking, regular exercise, frequency of toothbrushing, periodontitis, occupation, and metabolic syndrome.

Model 3 was adjusted for age, sex, BMI, current smoking, alcohol drinking, regular exercise, frequency of toothbrushing, periodontitis, occupation, metabolic syndrome, education, or income.

**Fig 2:**
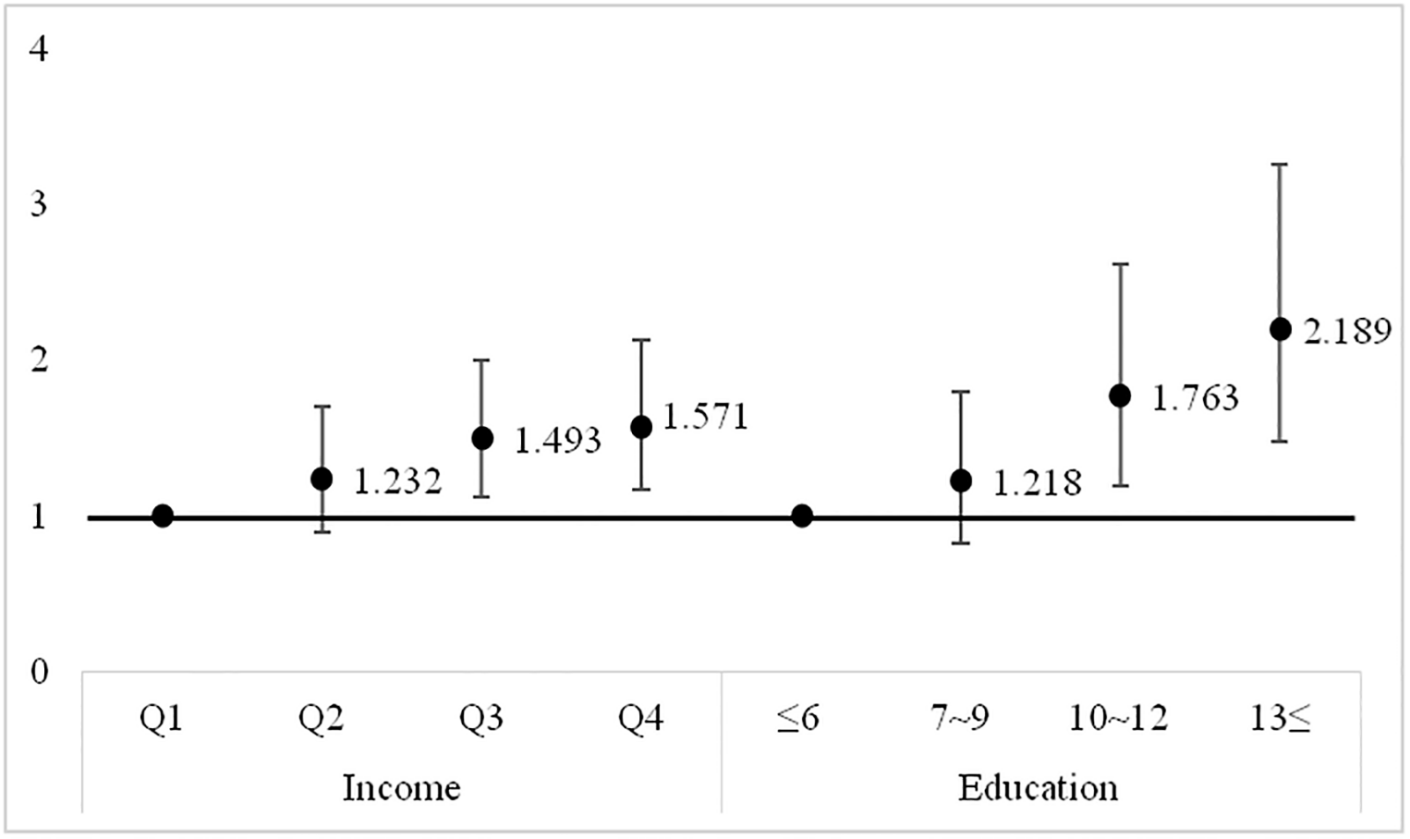
Multivariate adjusted odds ratios for having ≥20 remaining teeth according to each of the four income and education levels. Both p for trend <0.001. This model adjusted for age, sex, BMI, current smoking, alcohol drinking, regularexercises, frequency of tooth brushing, periodontitis, occupation, metabolic syndrome, education, and income.

Fig 2 shows the multivariate adjusted ORs for having ≥20 remaining teeth according to each of the four income and education levels. Regarding the reference categories (income Q1 and education ≤6 years), the ORs for ≥20 remaining teeth increased as income and education levels increased after adjusting for all covariates (OR = 1.49 and OR = 1.57 in incomes Q3 and Q4, respectively, and OR = 1.76 and OR = 2.18 in education levels 10–12yrs and ≥13yrs, respectively). 95% confidence interval.

## Discussion

In this study, the number of remaining teeth was significantly higher among participants in the upper income and education quartiles. Also, the 20 or more teeth group had a significantly higher percentage of people who were currently employed, had a regular exercise habit, had a lower WC, and lower rates of metabolic syndrome and periodontitis than the less than 20 teeth group. Income and education levels were higher in people with good oral-health behaviors, such as toothbrushing more than three times per day and receiving regular dental examinations.

Tooth loss results from various factors, including general health behaviors, medical health status, and SES [17, 22, 33–38]. Low SES was associated with increased tooth loss among elderly Americans [17], while increasing education level was found to be inversely associated with tooth loss in a US cohort study [38]. In a Brazilian study, low and middle SES were associated with more tooth loss compared with high SES [22, 39]. Some studies found that tooth loss may be a manifestation of socio-economic inequalities at the individual level and across ethnicities [37, 40–42]. In the elderly American population, poverty and minority race/ethnicity were significantly associated with oral-health disparities [17]. Hispanic and black adults were less likely to have routine dental examinations than non-Hispanic white adults, and they were at increased risk for poor oral health [17]. In affluent areas, disparities in tooth retention were negligible, but in poor neighborhoods, substantial variation in tooth retention between individuals was found based on their level of income. Living in a low SES neighborhood or not receiving regular dental care was associated with tooth loss [22, 23]. However, other studies found contrary associations or no association between tooth loss and SES, and that association patterns differed according to ethnicity [21–23, 43]. Poverty and education are not associated with number of missing teeth among Hispanic or black adults, while non–Hispanic white adults with lower SES presented with greater tooth loss [17]. Susin et al. reported that having four or more missing teeth was significantly associated with number of teeth with caries/fillings in a young Brazilian population [44]. Early oral-health education is warranted to save natural teeth as well as to improve general health behaviors.

The mechanism underlying the association between tooth loss and SES is general health behavior. Elderly Australians with a history of smoking had higher odds for tooth loss, but the risk declined as time since smoking cessation increased [33]. Other studies also showed that smoking was associated with tooth loss [39, 44]. Klein et al. have found that smoking and heavy alcohol consumption were associated with tooth loss [38]. In general, these poor health behaviors are common in subjects with low SES [45], while higher SES subjects exercised general health behaviors that promoted tooth maintenance, such as more frequent toothbrushing [1, 46]. Many previous studies showed an association between oral health and SES [15–17, 21] and, in this study, subjects with high SES had good oral health and received regular oral examinations. Use of fluoridated toothpaste and toothbrushing were associated with more remaining teeth in Lithuania [47], and in Denmark, toothbrushing more than two times per day was associated with more routine dental visits, more consistent dental care, and higher education level [48]. In a Taiwanese study, it was observed that regular toothbrushing, use of extra oral hygiene products such as dental floss and mouthwash, and receiving dental scaling were associated with having more remaining teeth [49]. It has also been shown that middle-aged Brazilian adults who had not visited a dentist in the last three years had a 33.5% increased risk for tooth loss [22]. Other researchers have shown that poor oral health behaviors were associated with increased prevalence of periodontitis [50] and metabolic syndrome [2, 16], which are also associated with increased systemic inflammation [51–53] due to cytokine production, such as tumor necrosis factor-α and interleukins-1 and -6 [54, 55], that also increased tooth loss.

There are several limitations in this study. First, this is a cross-sectional study and we cannot identify causal relationships. Second, we did not evaluate inflammatory markers as a mechanism of this relationship. Third, we could not obtain further information about individual causes of tooth loss from the subjects. However, this study has some strength. The subjects of this study are a representative sample of the whole Korean population using the KNHANES. Korea is a relatively ethnically homogenous population therefore, we could exclude racial/ethnical biases in the relationship between SES and number of remaining teeth. Also, we adjusted for several covariates that are known to be associated with number of remaining teeth from many previous studies.

In conclusion, low SES is associated with significant tooth loss. A greater proportion of subjects with higher SES presented with good health behaviors and greater numbers of remaining teeth than did low SES subjects. Oral healthcare professionals should educate their patients regarding the importance of proper oral-health behaviors for preventing tooth loss, especially their low SES patients. Further prospective studies are needed to evaluate the mechanisms and the relationships between SES and tooth loss, and to develop interventions to preserve remaining teeth.
